# SARS-CoV-2 vaccination in cardiothoracic organ transplant recipients: effective strategies wanted

**DOI:** 10.1007/s00392-021-01876-1

**Published:** 2021-07-09

**Authors:** Sebastian Ewen, Frank Neumann, Jörg T. Bittenbring, Wolfgang von Scheidt, Michael Böhm

**Affiliations:** 1 Klinik für Innere Medizin III, Kardiologie, Angiologie und Internistische Intensivmedizin, Universitätsklinikum des Saarlandes und Medizinische Fakultät der Universität des Saarlandes, Kirrberger Str. 100, 66421 Homburg, Saar Deutschland; 2 Klinik für Innere Medizin I, Onkologie, Hämatologie, Klinische Immunologie und Rheumatologie, Universitätsklinikum des Saarlandes und Medizinische Fakultät der Universität des Saarlandes, Homburg, Saar Deutschland; 3 I. Medizinische Klinik, Kardiologie, Pneumologie, Internistische Intensivmedizin, Endokrinologie, Universitätsklinikum Augsburg, Augsburg, Deutschland

**Keywords:** SARS-CoV-2, Vaccination, Cardiothoracic organ transplant recipients

Since end of 2019, the severe acute respiratory syndrome coronavirus 2 (SARS-CoV-2) has spread from Wuhan, China to the rest of the world, leading to more than 160 million confirmed cases of coronavirus disease 2019 (COVID-19) with more than 3.3 million deaths worldwide [1]. COVID-19 results in a spectrum of clinical manifestations ranging from asymptomatic to critical illness including acute respiratory distress syndrome (ARDS) and multiorgan failure, which are associated with high morbidity and mortality. Factors that increase risk for an adverse disease course include older age and the presence of comorbidities such as diabetes, hypertension, chronic kidney disease, morbid obesity, coronary heart disease, and chronic lung disease [2]. Solid organ transplant (SOT) patients are considered to be at high risk for complications from COVID-19 because of the high prevalence of the comorbidities that have been established as risk factors for severe disease, as well as a higher risk of infection due to their immunosuppressed status [3, 4]. Current data on the clinical course of COVID-19 in immunocompromised patients are limited.

Vaccination against COVID-19 may be an effective intervention in fighting the pandemic. Several SARS-CoV-2 vaccines have been approved for administration in various countries and are available to select populations based on local recommendations and regulations. Current efficacy data from randomized clinical trials in immunocompetent recipients are variable as noted in Table 1, but thus far consistently demonstrate close to 100% protection against severe COVID-19-related intensive care hospitalization or death [5]. Observational “real-world” findings from Israel, including 596,618 vaccinated persons matched to unvaccinated controls, demonstrated an 87% reduction in COVID-19-related hospitalization following administration of two doses of the Pfizer-BioNTech vaccine [6]. Safety and efficacy data for SARS-CoV-2 vaccines in transplant recipients are limited thus far, as transplant recipients were not included in the already published randomized vaccine trials [5]. However, guidelines regarding COVID-19 vaccination in patients with thoracic transplantation have been released by the International Society for Heart and Lung Transplantation (ISHLT) and the American Society for Transplantation (AST) in a combined statement [7], as well as by the European Society of Cardiology (ESC) [8], unequivocally recommending vaccination at least 1 month after transplant surgery.

**Table 1:** SARS-CoV-2 vaccinations available globally, as of May 10, 2021 (Adapted from [5])

Manufacturer	Type	Vaccine efficacy reported in clinical trials (%)	Prevention of severe disease leading to death in clinical trials (%)
Pfizer/BioNTech	mRNA	95	100
Moderna	mRNA	94.5	100
Sputnik V	Ad26/Ad5	91.4	100
AstraZeneca	ChAdOx1	60–85	100
Johnson & Johnson	Ad26	57–72	100
Cansino	Ad5	66	100
Sinopharm	Inactivated	79.6–86	100
Sinovac	Inactivated	50.1	100
Novavax	Protein	86–96	100

In this issue of the Journal, Schramm and colleagues [9] report on a prospective cohort study of the immune response after administration of the BNT162b2 vaccine in 50 cardiothoracic (heart (*n* = 42), lung (*n* = 7) or heart–lung (*n* = 1)) transplant recipients from 5 German transplant centers 10–36 months after transplantation, compared to 50 healthy staff members similarly immunized with the same messenger RNA (mRNA) vaccine. Antibody titers, functional inhibitory capacity of neutralizing antibodies, and T-cell response (interferon-γ release) were measured via three separate tests. Forty-six (92%) of the transplant recipients did not show any humoral or T-cell response 21 days after completion of the second vaccine dose [9]. In contrast, humoral vaccination success (IgG antibodies and neutralizing antibodies) in healthy controls was seen in 49 subjects (98%) after the first vaccine dose, and in 100% after the second vaccine dose [9]. Additionally, healthy controls developed a significantly higher positive T-cell response (interferon-γ release) compared to transplant recipients (80% vs 16%) [9]. It is concluded that the BNT162b2 vaccine does not confer vaccination success against SARS-CoV-2 in the vast majority of cardiothoracic transplant recipients.

The presented data indicate that patients receiving immunosuppression are less likely to respond to mRNA-based SARS-CoV-2 vaccination compared to immunocompetent participants included in SARS-CoV-2 vaccine trials [10]. Although data regarding the new vaccine technology using mRNA in transplant recipients are scarce, it is known that patients who underwent a transplant do not develop optimal immune responses following many vaccines (e.g., influenza vaccination) [11]. The reasons for this are multifactorial but may include (i) the time since transplantation, (ii) the amount and nature of immunosuppression and (iii) the occurrence of concomitant allograft rejection that interferes with the production of the requisite neutralizing antibodies [12]. A study of COVID-19 vaccine responses among kidney transplant recipients indicated that all 25 members of the control group developed a positive humoral immune response to the viral spike proteins, compared with only 37.5% out of 136 transplant recipients who had a positive serology test [13]. A prospective cohort study including 658 SOT recipients (97 heart recipients and 71 patients who underwent lung transplantation) across the US showed that only a low proportion (15%) of transplant patients mounted a positive antibody response after the first vaccine dose, which increased to 54% of transplant recipients after dose 2 [12]. Although this study demonstrates an increase in antibody responses in transplant patients after the second vaccine dose compared with the first, data indicates, in line with the investigation presented herein, that a substantial proportion of transplant patients are likely to remain at risk for COVID-19 despite being administered a full dose of an mRNA-based vaccine. Currently, there are not enough comparative studies in transplant patients evaluating the differences in immune response to the various COVID-19 vaccine types already developed. Therefore, further studies are urgently needed to address interventions including different types of vaccination, additional booster doses, optimization of t-cell responses of current vaccines or immunosuppression modulation to improve vaccine responses in this high-risk population. Larger, multicenter studies also need to be performed to disentangle the group of non-responders from the group who would benefit from traditional dosing. It remains unclear whether a lack of antibody response after COVID-19 vaccination is associated with a higher risk of infection or a worse course of the disease after infection.

Although the exact impact of chronic immunosuppression on COVID-19 outcomes remains ambiguous as of today, its role seems to be highly relevant as host inflammatory responses appear to constitute an important cause of associated organ injury [3]. As the manifestations of severe COVID-19, including ARDS and organ dysfunction, may be propagated by a proinflammatory state due to cytokine release syndrome, immunosuppressive therapy could potentially mitigate some of these effects and thereby help prevent severe complications in SOT patients [3]. One multicenter study comparing ICU COVID-19-positive SOT recipients with matched controls found no difference in rates of ARDS or death between groups [3]. Although some smaller single-center studies have shown excess mortality in patients who underwent a transplant, many were conducted in regions with overwhelmed hospital systems at the time of patient presentation [14]. In the case of vulnerable, immunocompromised patients with a severe disease course, passive immunization against COVID-19 using convalescent sera or engineered antibody cocktails might be an option, barring circulating resistant variants. However, in 228 randomized hospitalized patients with severe COVID-19 pneumonia, no significant differences in clinical status or mortality were detected after 30 days between patients treated with convalescent plasma and those who received placebo [15].

COVID-19 will likely circulate for many years, if not indefinitely. Based on the presented findings, routinely testing for antibody response (or even T-cell response) in transplant recipients following COVID-19 vaccination should be strongly recommended to prevent false security in this high-risk group. All transplant recipients have to be counseled to continue practicing protective measures including mask use, hand hygiene, and social distancing even if they are vaccinated. As with other very contagious viral pathogens such as measles, real protection of the immunocompromised could only be possible by vaccinating relatives and all fellow human beings to achieve “herd immunity”. The ultimate question that remains is whether it should continue to be recommended that all patients be vaccinated against COVID-19 after heart or lung transplants—as of today, the answer is YES! But more effective, yet unclear strategies are urgently needed.
